# Supplementary material for: Pangenome analysis of transposable element insertion polymorphisms reveals features underlying cold tolerance in rice
Source: Nat Commun. 2025 Aug 16;16:7634. doi: 10.1038/s41467-025-62887-4 (PMC12357961; doi:10.1038/s41467-025-62887-4)
Supplement: Supplementary file 1 — Supplementary Information [file 41467_2025_62887_MOESM1_ESM.pdf]

Pangenome analysis of transposable element insertion polymorphisms reveals features underlying cold tolerance in rice

Qian *et al.*

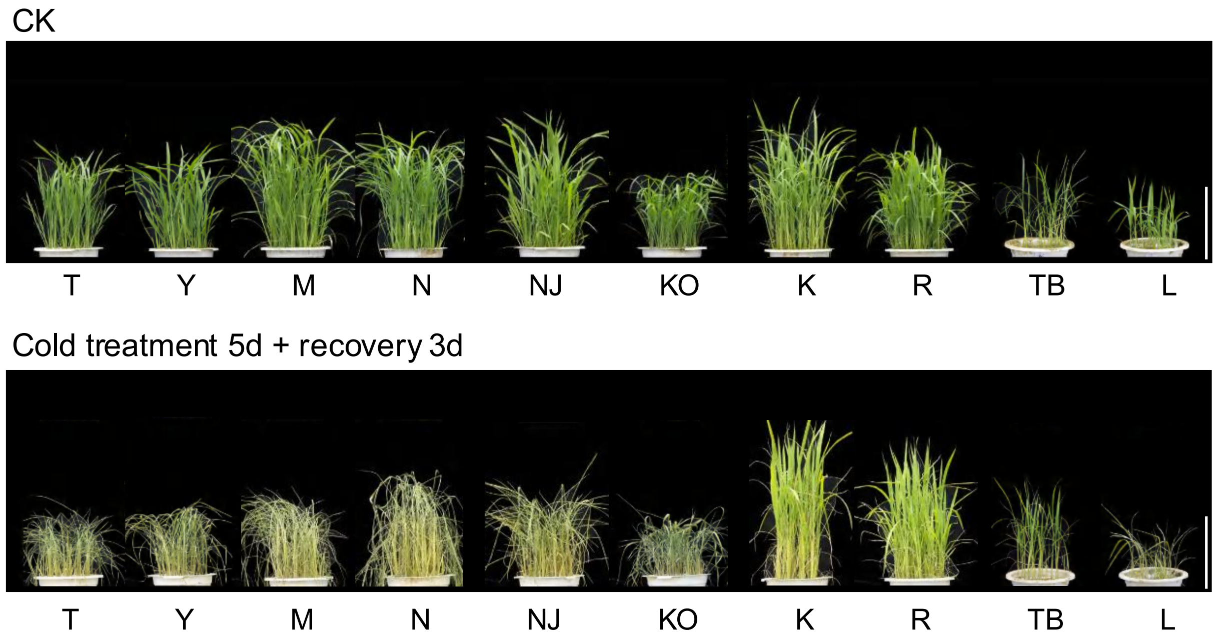

Supplementary Figure 1. Plant morphology of 10 rice accessions under normal and cold treatment conditions[1]. Scale bar: 20 cm.

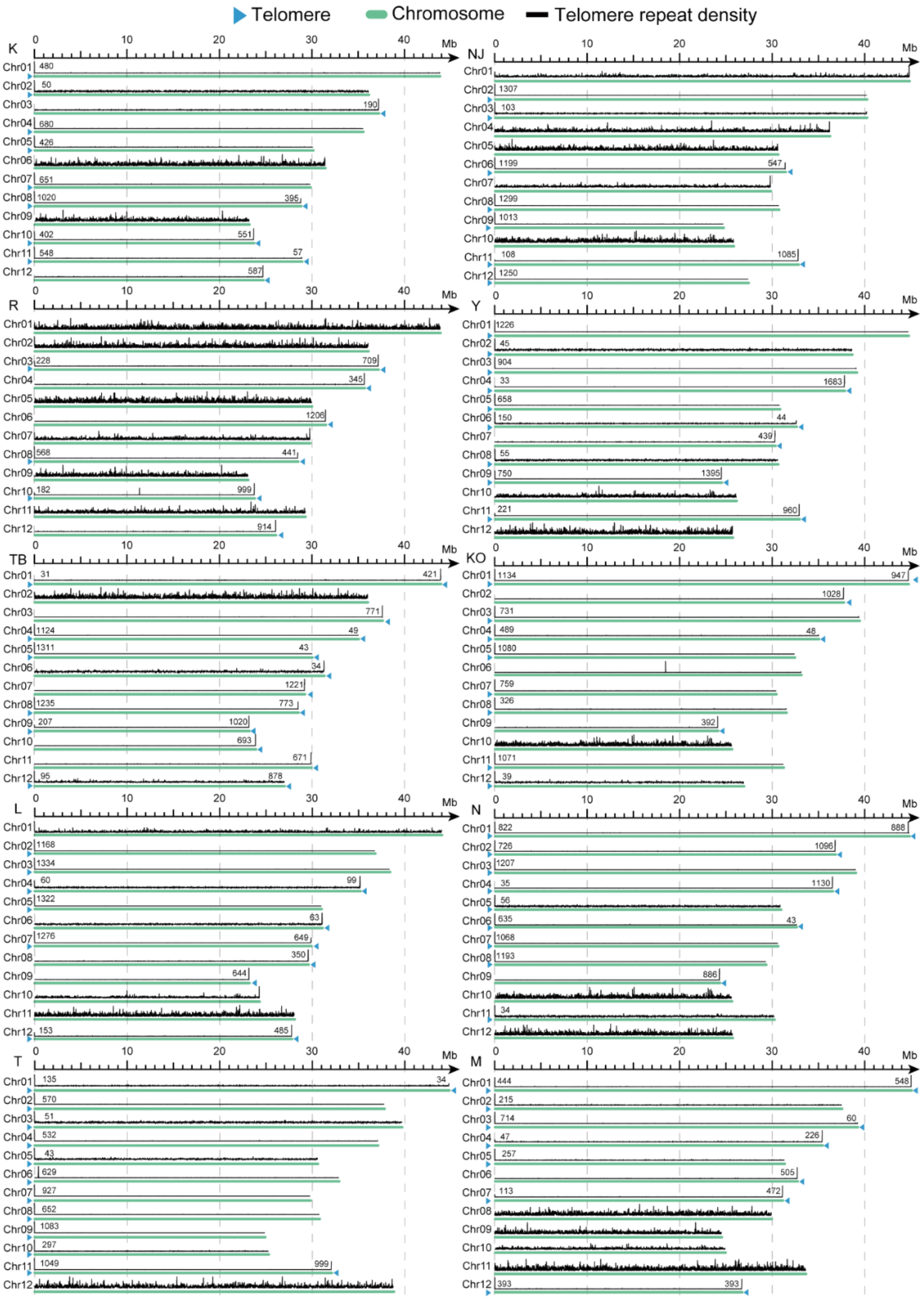

Supplementary Figure 2. The distribution of telomeres in 10 rice accessions. The blue triangles at both ends of the chromosome represent telomeres, and the numbers indicate the repeat counts of the telomeric repeat sequences.

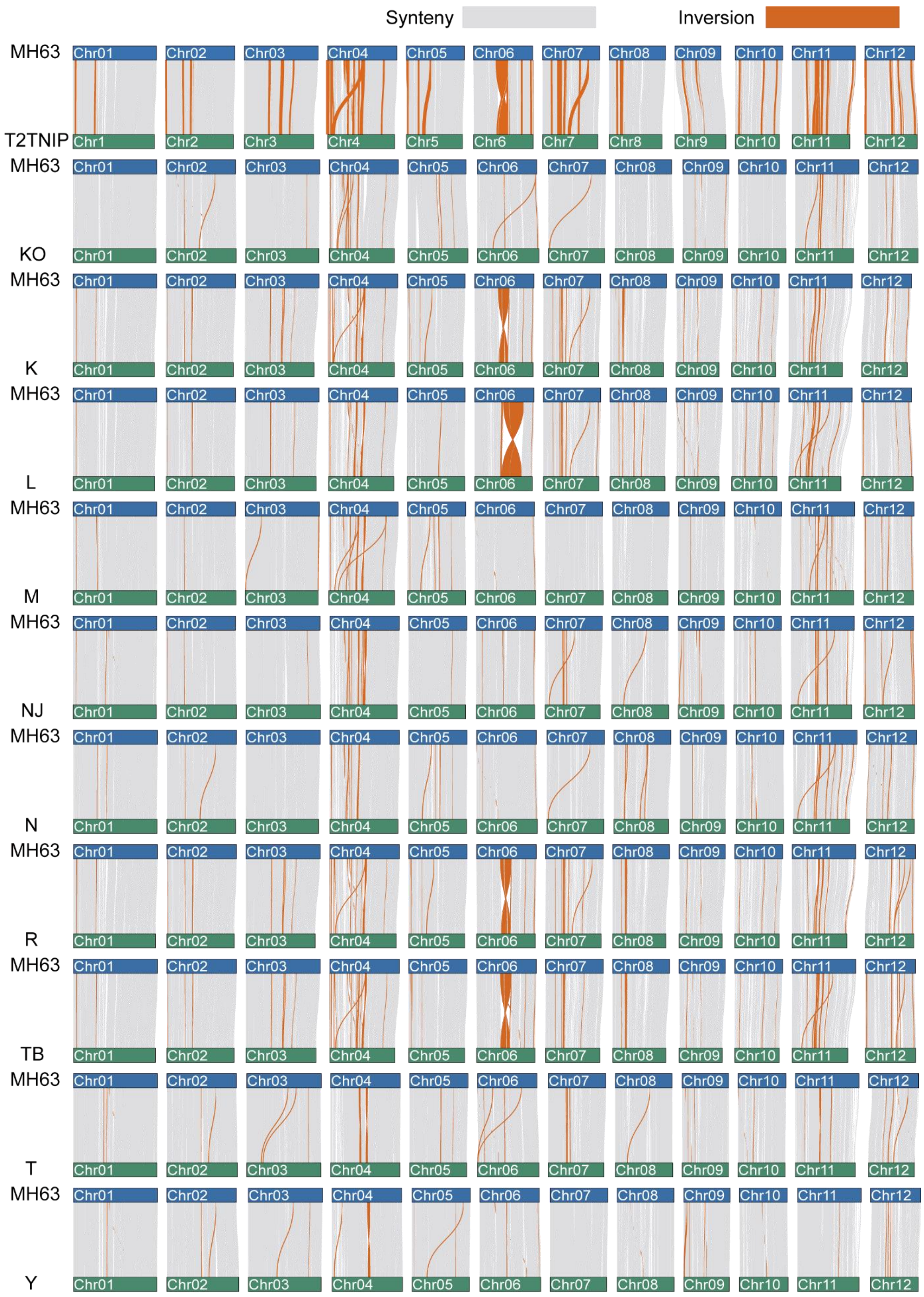

Supplementary Figure 3. Comparative syntenic analysis between 11 rice genomes and reference genome MH63.

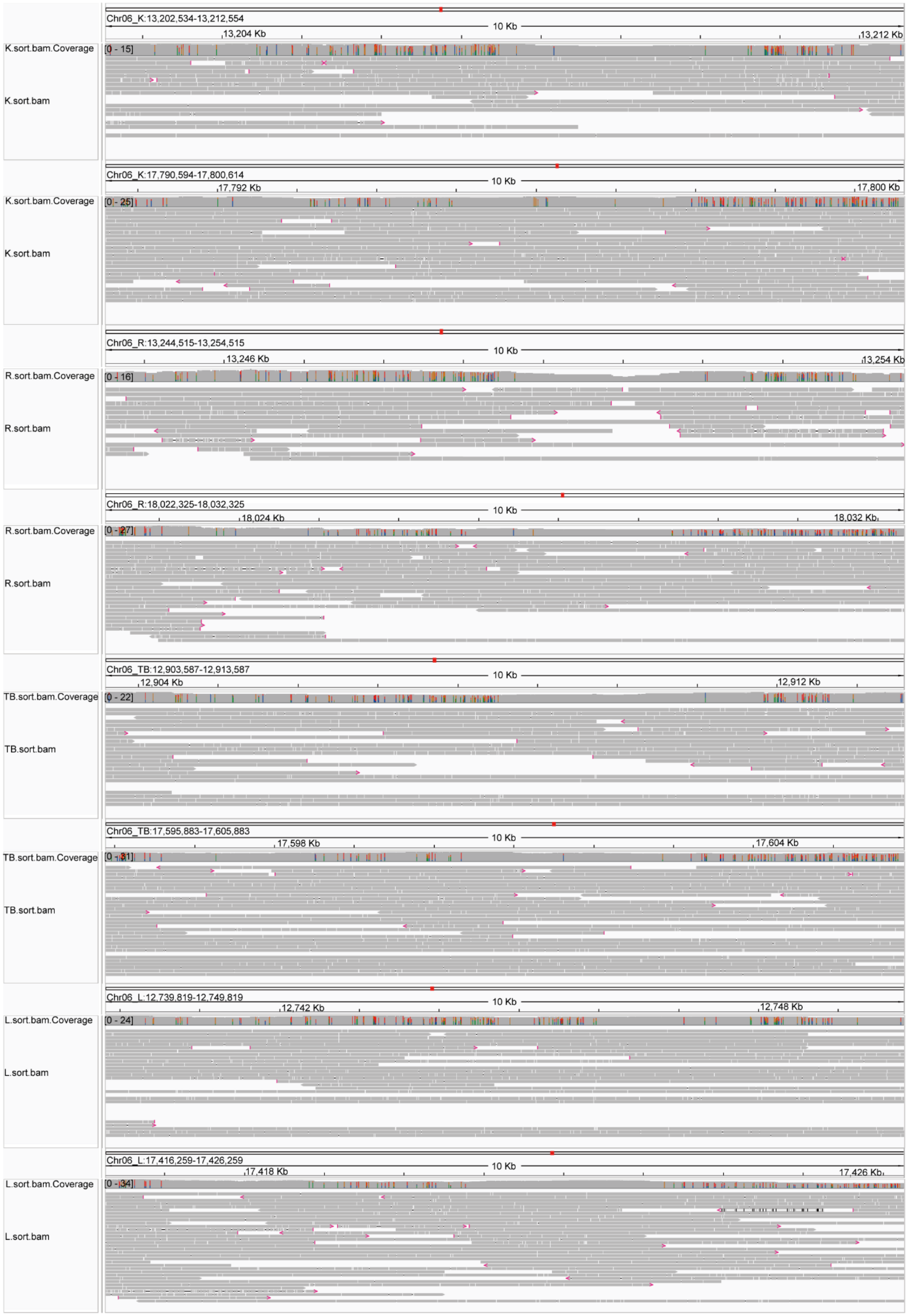

Supplementary Figure 4. The read alignment near the inversion on chromosome 6 of *japonica* rice.

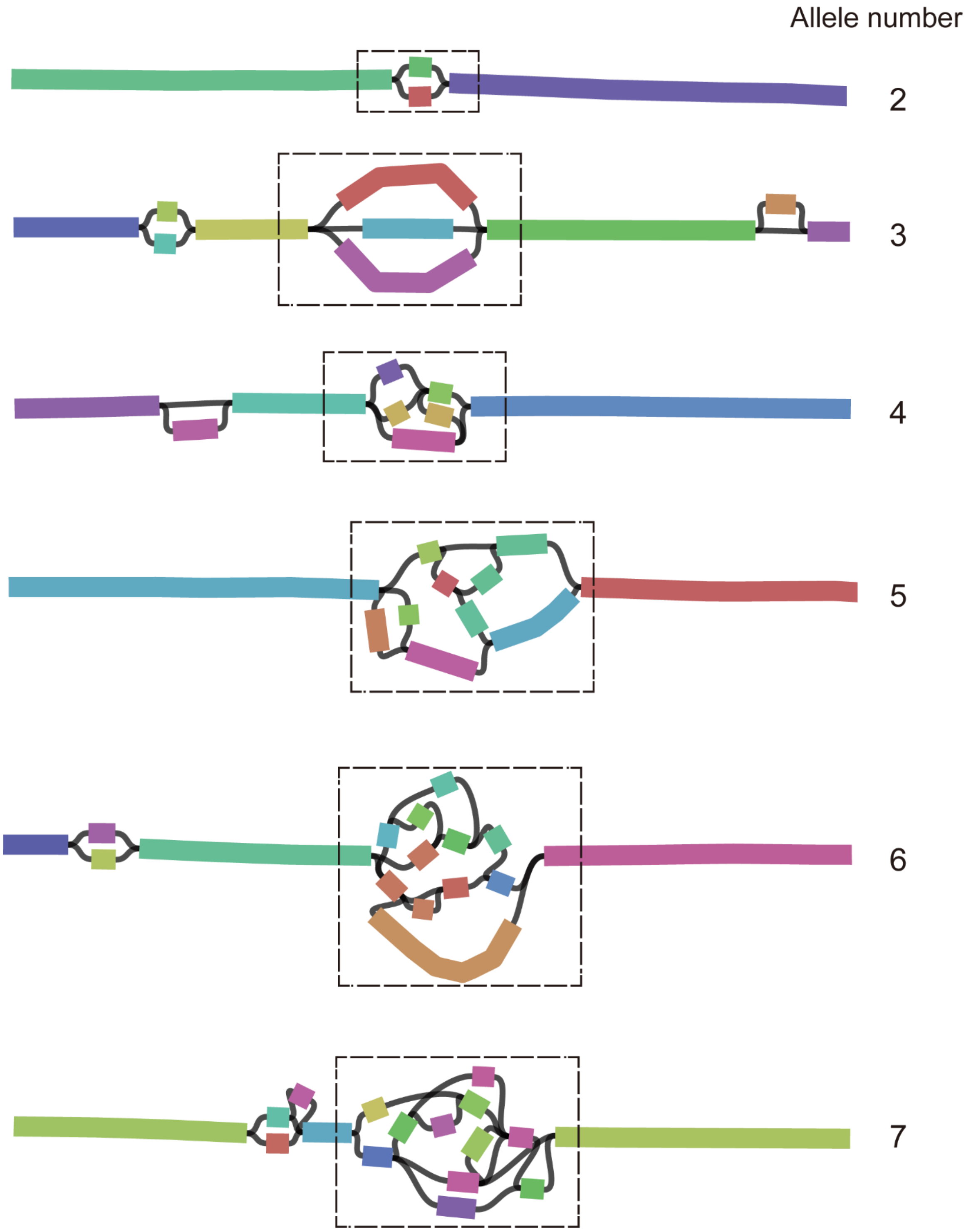

Supplementary Figure 5. Schematic diagram and number of different SV types.

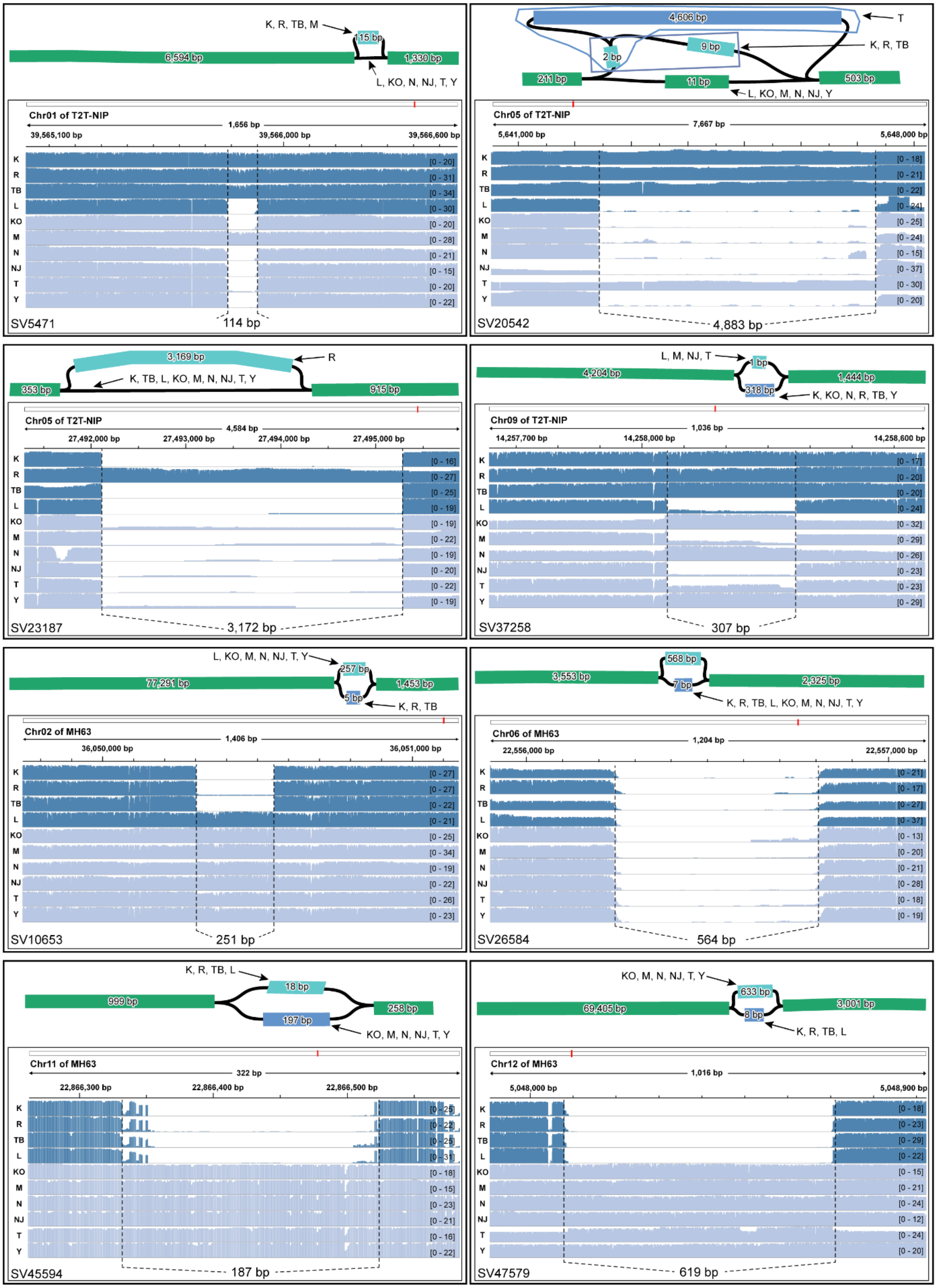

Supplementary Figure 6. Validation of TIP genotyping based on read mapping.

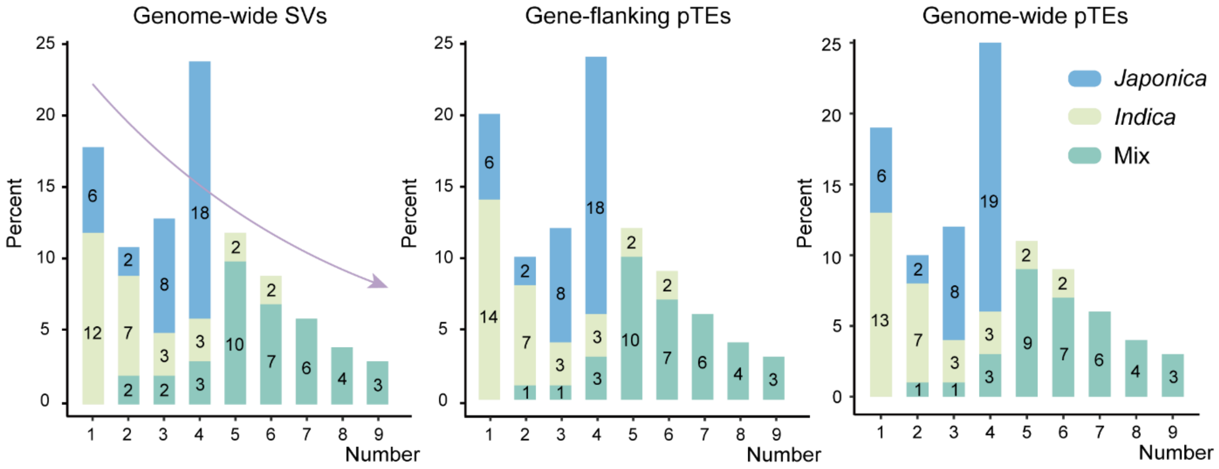

Supplementary Figure 7. The distribution of gene-flanking pTEs, genome-wide pTEs, and genome-wide SVs.

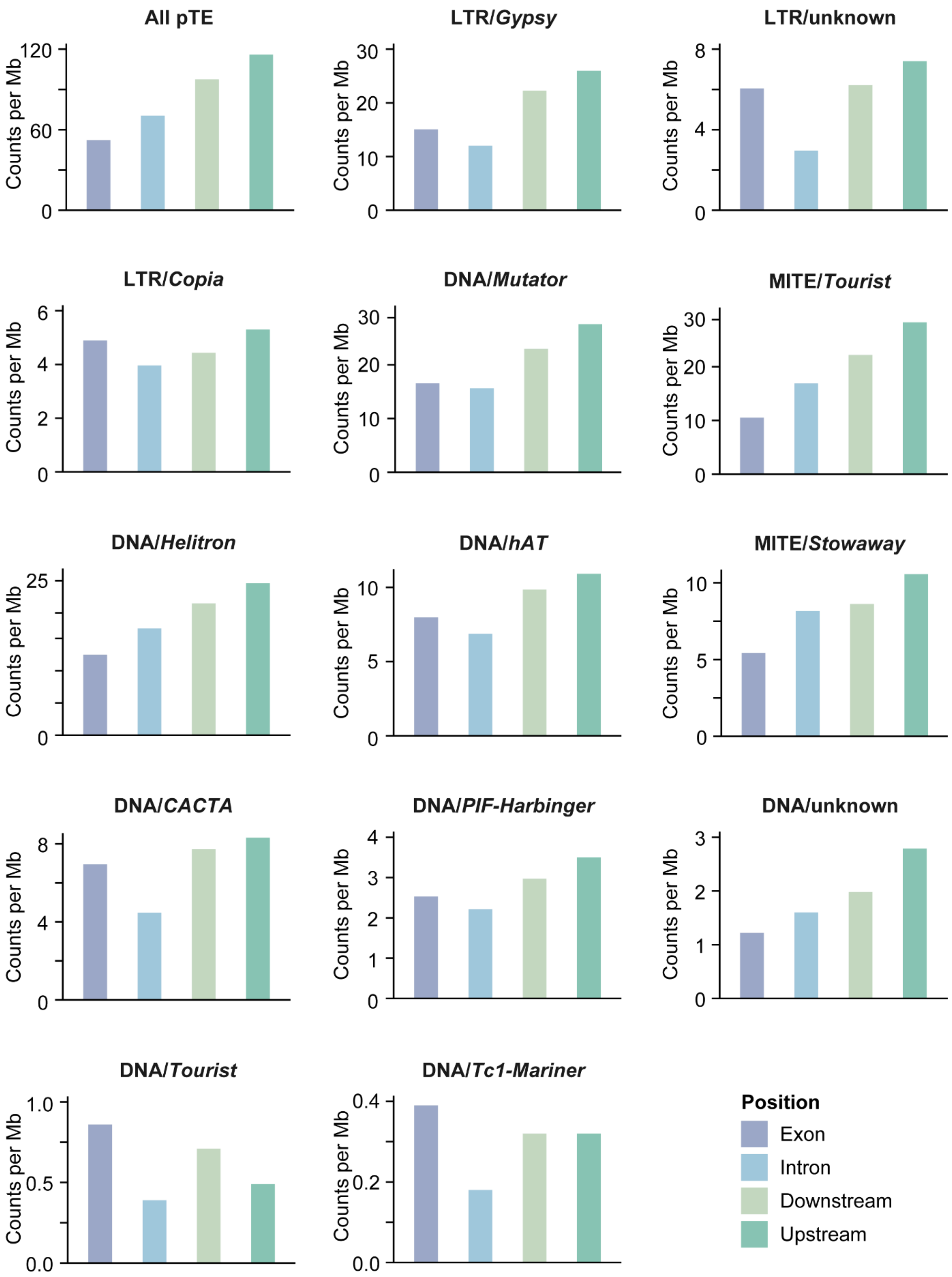

Supplementary Figure 8. Bar plot showing the pTE insertion densities across different sequence regions. Source data are provided as a Source Data file.

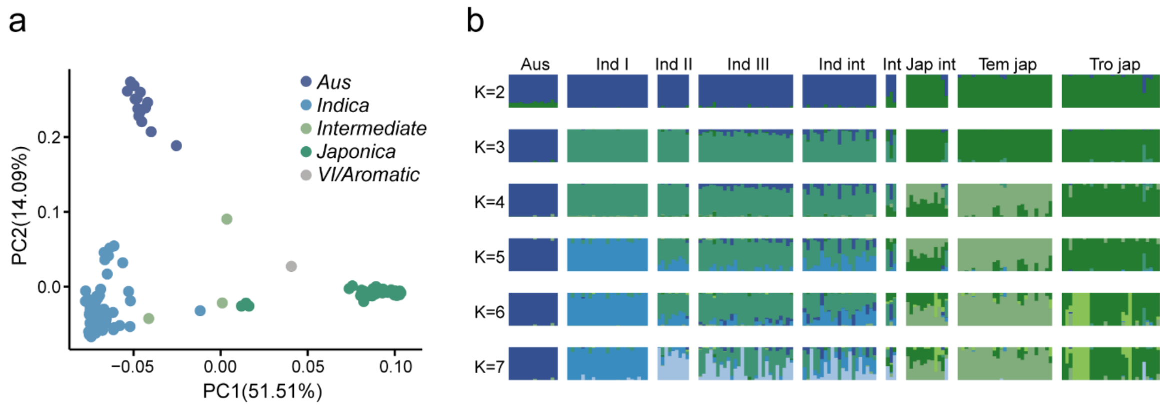

Supplementary Figure 9. Population analysis of 165 rice accessions based on SNPs. a, Principal component analysis (PCA) plot of 165 rice accessions based on SNPs. **b**, Population structure analysis of 165 rice accessions based on SNPs.

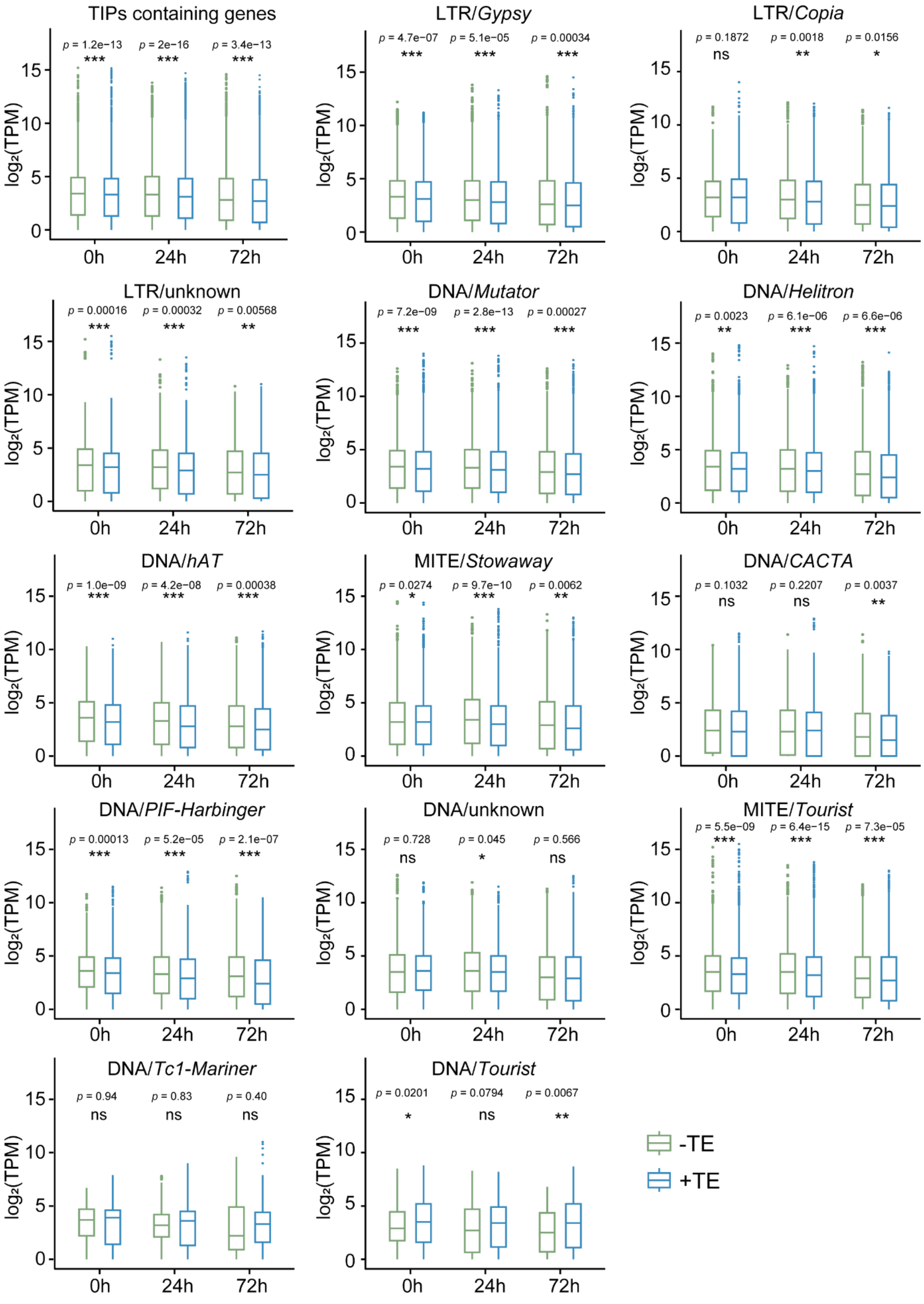

Supplementary Figure 10. Box plot of gene expression for TIPs-containing genes and genes with different types of pTE insertions. Statistical analysis of these data was performed using a two-tailed Wilcoxon test (***$P < 0.001$, **$P < 0.01$, ns: $P > 0.05$).

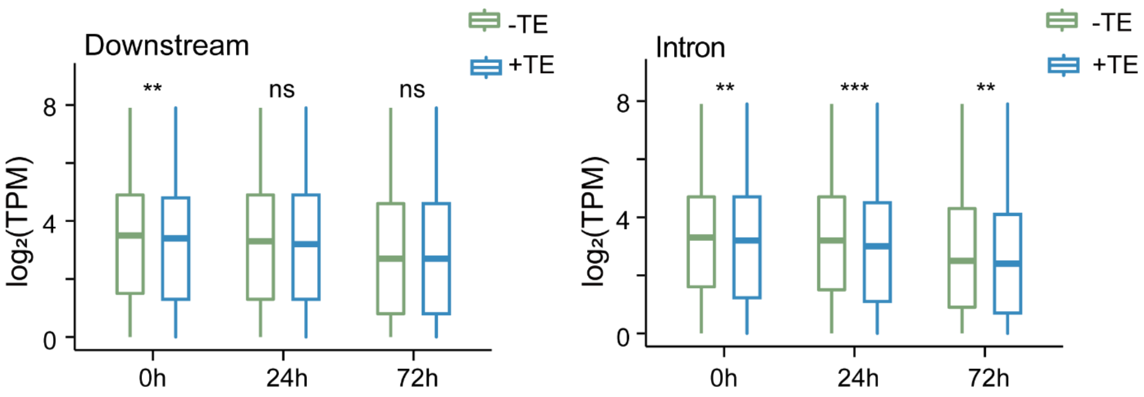

Supplementary Figure 11. The impact of pTE insertions on gene expression levels at different cold treatment time points. pTEs in different rice varieties are categorized into two groups: those with TE (+TE) and those without TE (-TE). "Downstream" indicates that a single pTE is located within 2 kb of the gene and inserted in the downstream region, while "Intron" signifies that a single pTE is also located within 2 kb of the gene but inserted within the intron. Statistical analysis of these data was performed using a two-tailed Wilcoxon test (***$P < 0.001$, **$P < 0.01$, ns: $P > 0.05$). Source data are provided as a Source Data file.

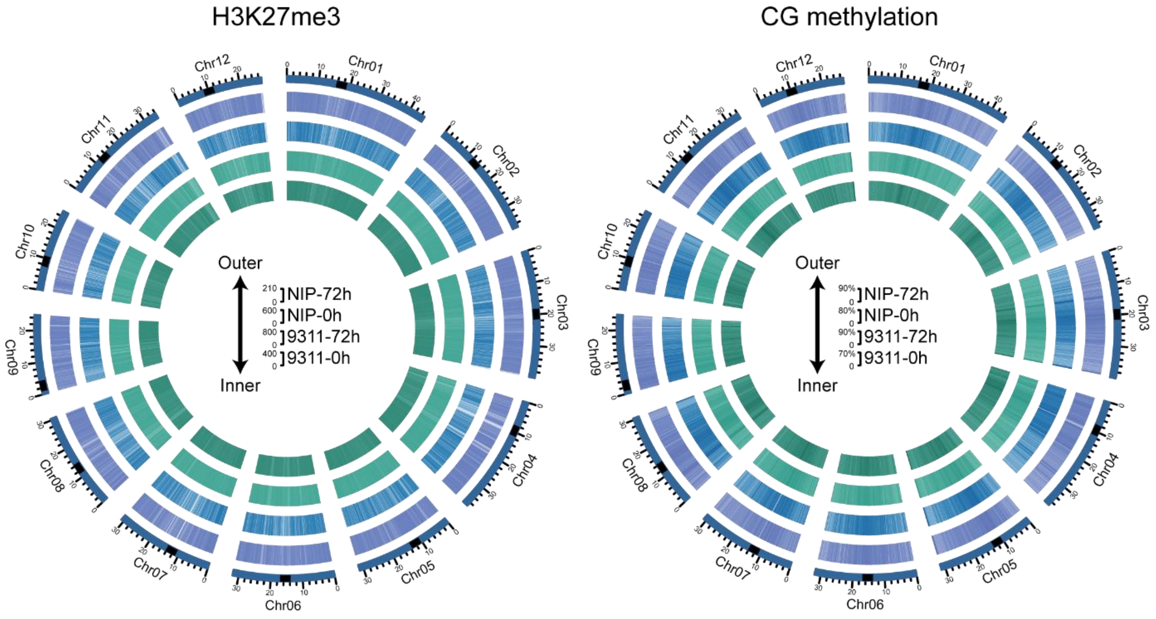

Supplementary Figure 12. Circos plot of methylation and H3K27me3 modifications in 9311 and Nipponbare under normal conditions and after cold treatment 72 hours. The black regions on each chromosome represent the centromeres.

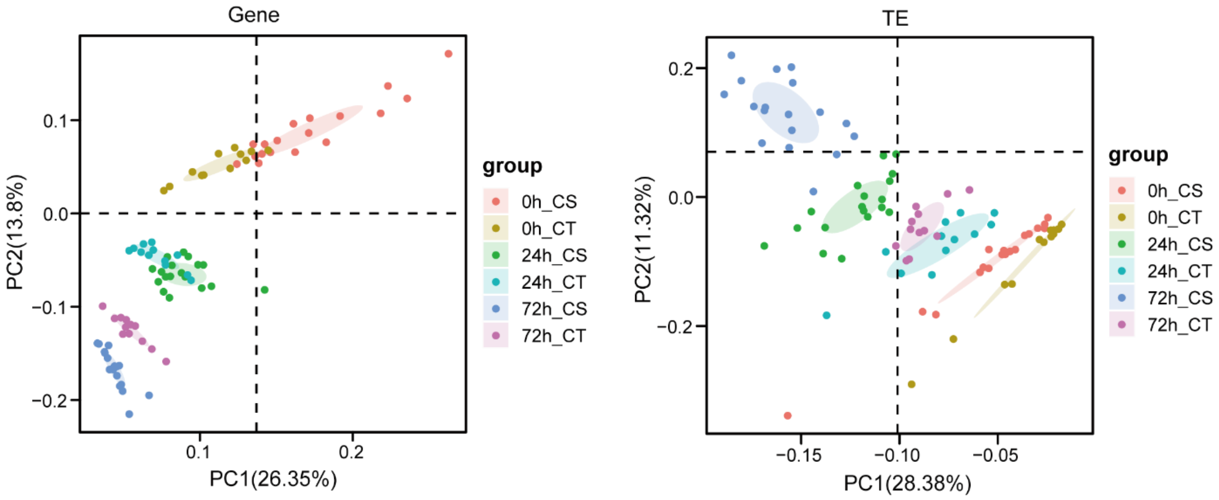

Supplementary Figure 13. PCA plot of gene and TE expression levels across different samples.

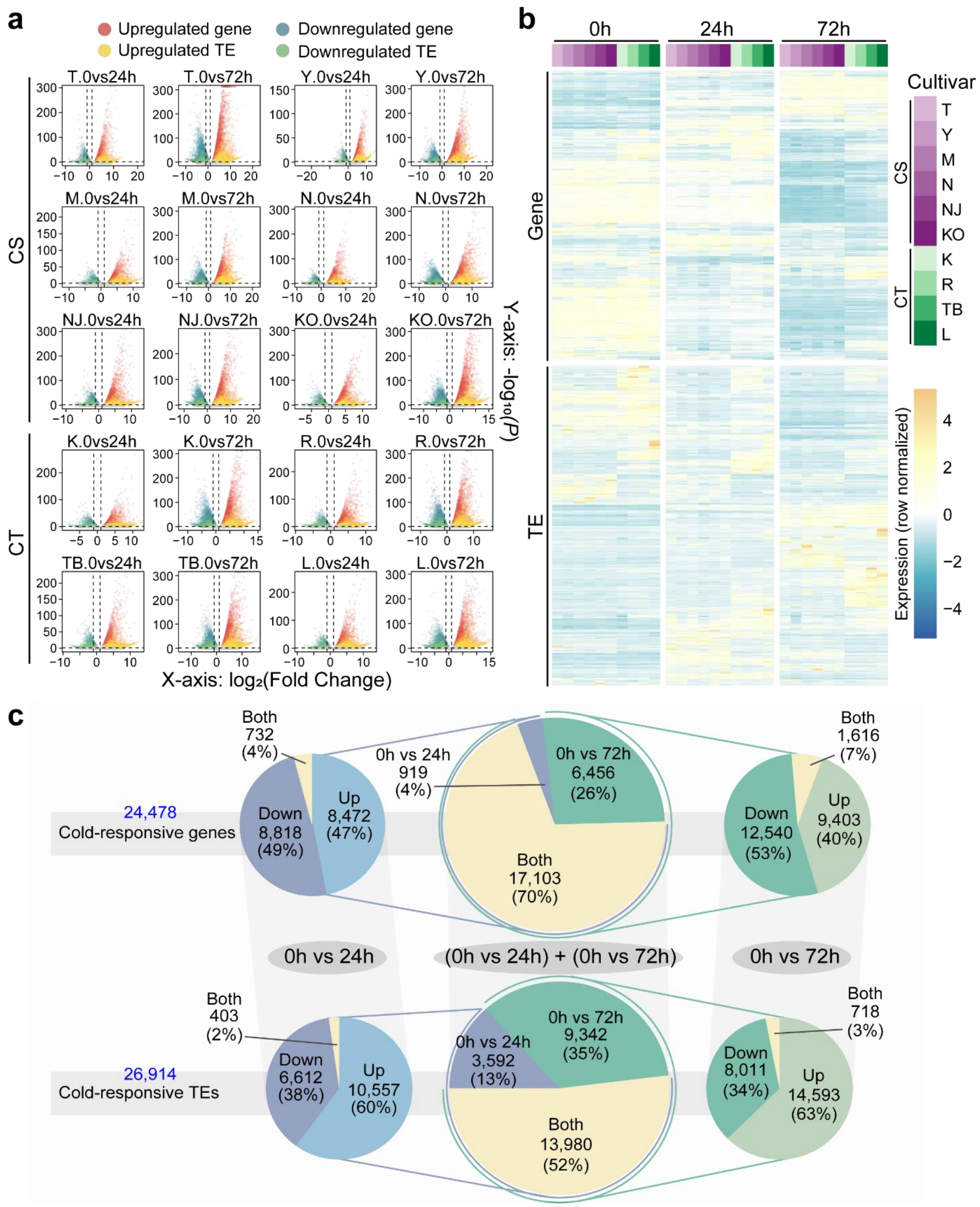

Supplementary Figure 14. Evaluation of expression differences across 10 rice accessions under various cold treatment conditions. a, Differentially expressed genes and TEs identified in each accession under cold treatments (0h vs. 24h and 0h vs. 72h). **b**, Expression patterns of all cold-responsive genes and cold-responsive TEs across 10 rice accessions under normal conditions (0h), cold treatment for 24 hours (24h), and cold treatment for 72 hours (72h). **c**, Pie plot of cold-responsive genes and TEs.

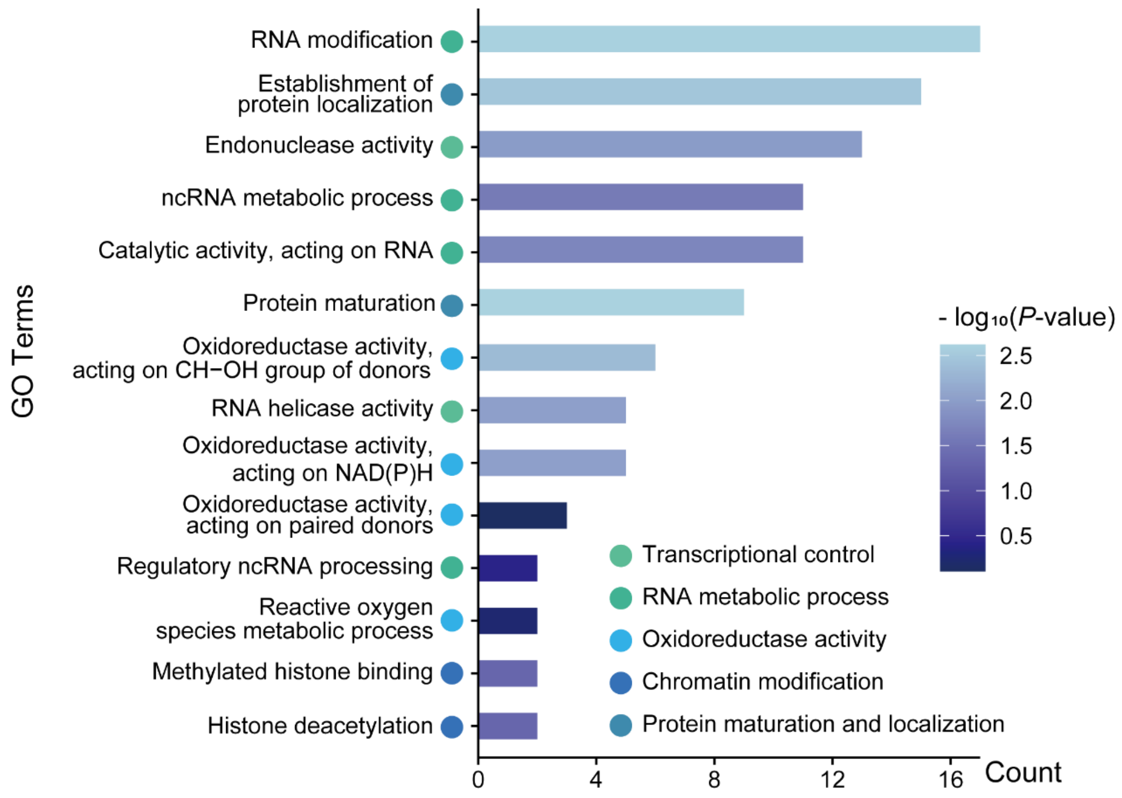

Supplementary Figure 15. GO enrichment analysis of coding genes associated with cis-TEs.

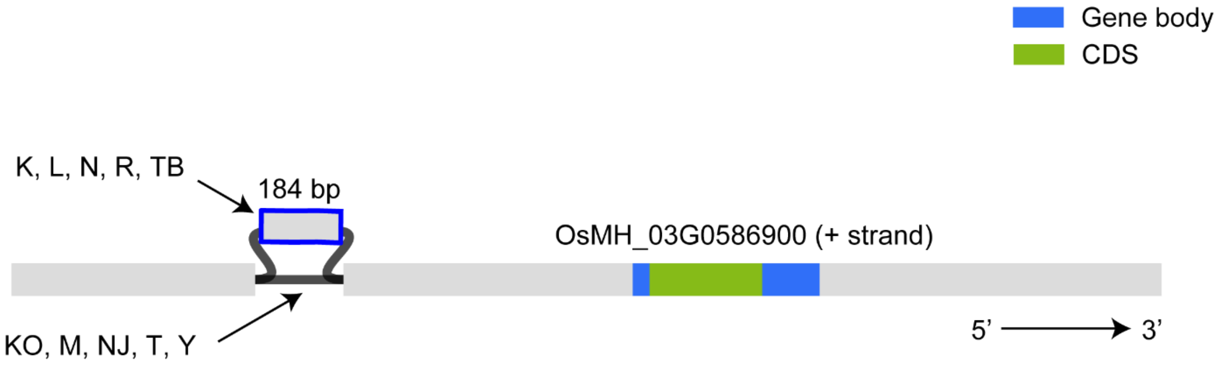

Supplementary Figure 16. Gene structure of OsMH_03G0586900 and the associated 184 bp insertion upstream 1,318 bp of the gene.

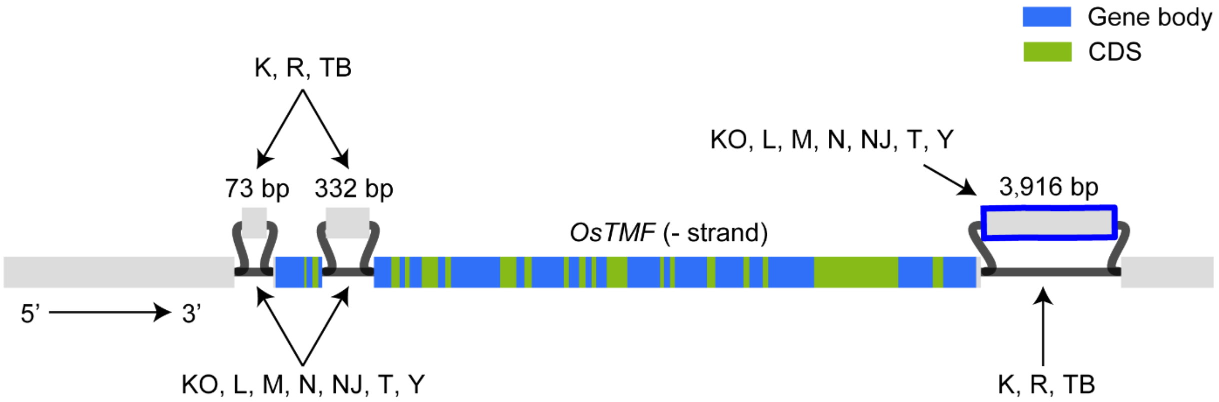

Supplementary Figure 17. Gene structure of *OsTMF* and the associated 3,916 bp pTE in the promoter region.

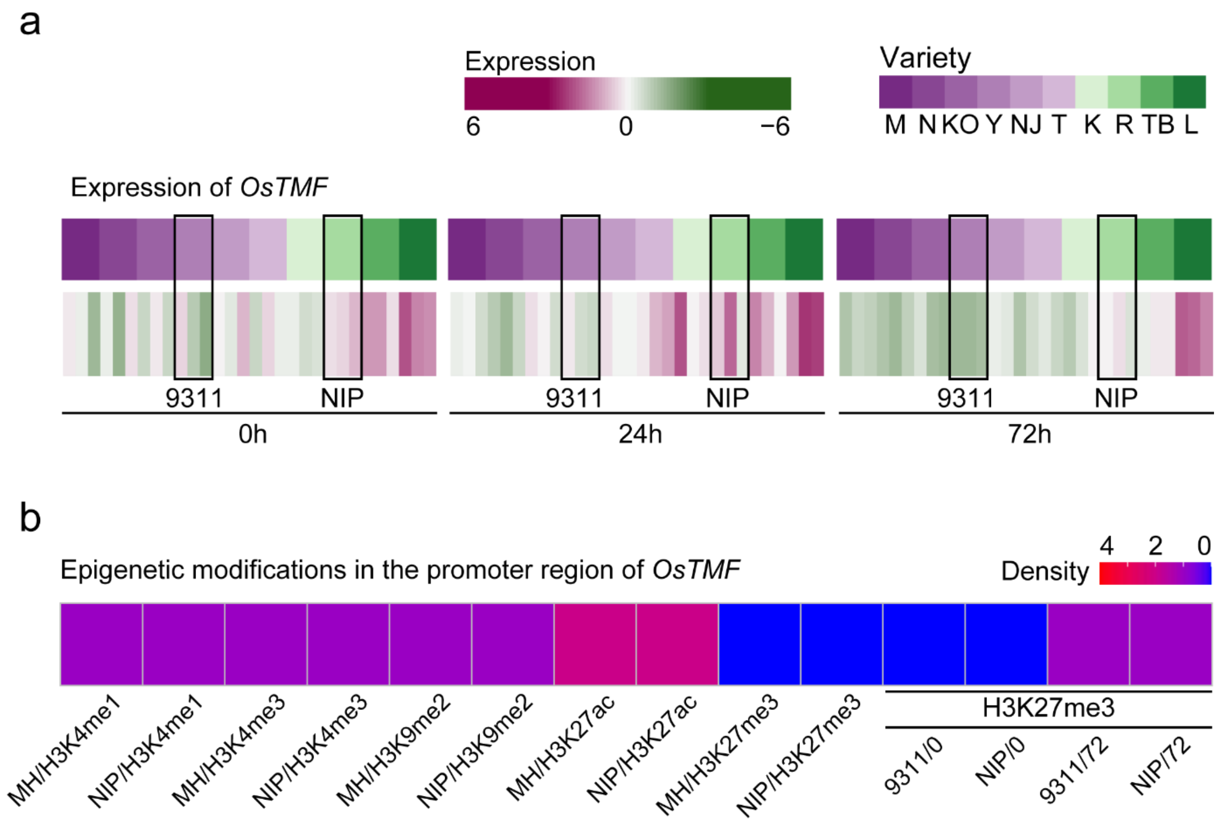

Supplementary Figure 18. Expression levels of *OsTMF* and histone modifications in the promoter region of *OsTMF* in 9311, MH63 and Nipponbare. a, Expression levels of *OsTMF* in different rice accessions at various cold treatment time points. **b**, Multiple histone modifications in the promoter region of *OsTMF* in Nipponbare and MH63, and H3K27me3 modifications in the promoter region of *OsTMF* in Nipponbare and 9311 before and after 72 hours of cold treatment.

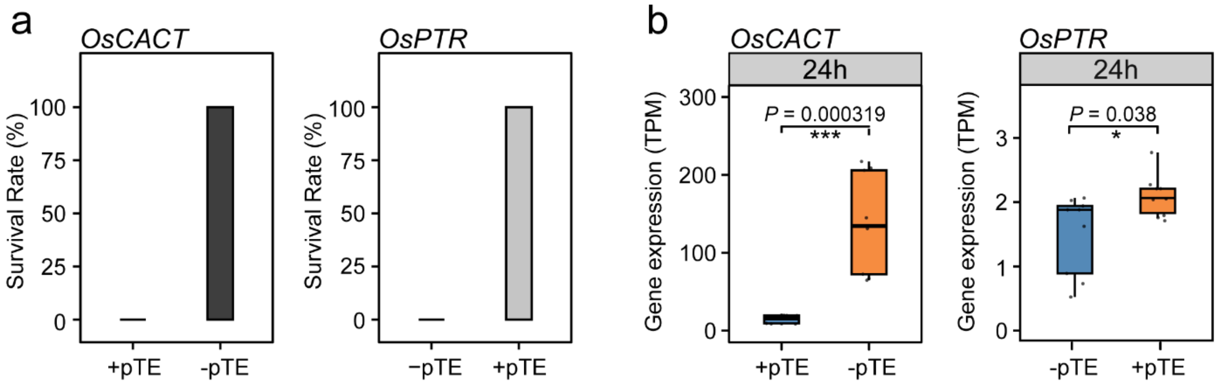

Supplementary Figure 19. Phenotypic and transcriptional differences of *OsCACT* and *OsPTR* between *japonica* and *indica* haplotypes under cold stress. a, Comparison of survival rates for different haplotypes of *OsCACT* and *OsPTR* under cold stress. **b**, Comparison of gene expression for different haplotypes of *OsCACT* and *OsPTR* under cold stress. Statistical analysis was performed using a two-tailed Wilcoxon test (**$P < 0.01$, ***$P < 0.001$). Source data are provided as a Source Data file.

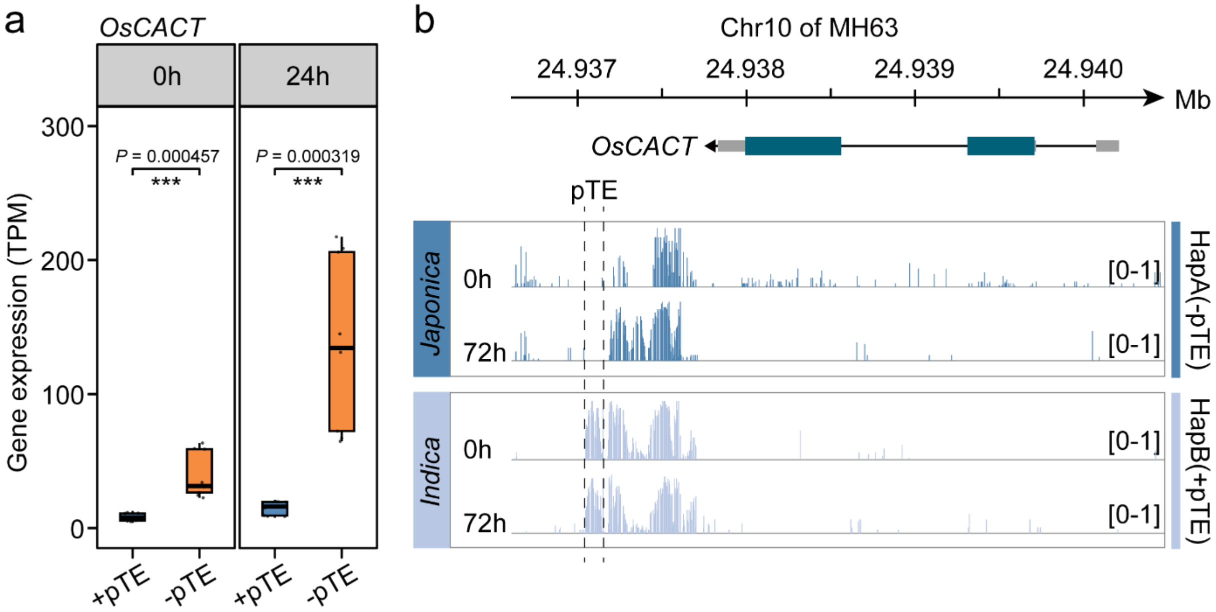

Supplementary Figure 20. Impact of pTE insertion and DNA methylation on *OsCACT* expression. a, *OsCACT* expression level in accessions with pTE (+pTE) or without pTE (-pTE). **b**, DNA methylation levels before and after cold treatment. The region between the two dashed lines represents the pTE. Statistical analysis was performed using a two-tailed Wilcoxon test (***P < 0.001). Source data are provided as a Source Data file.

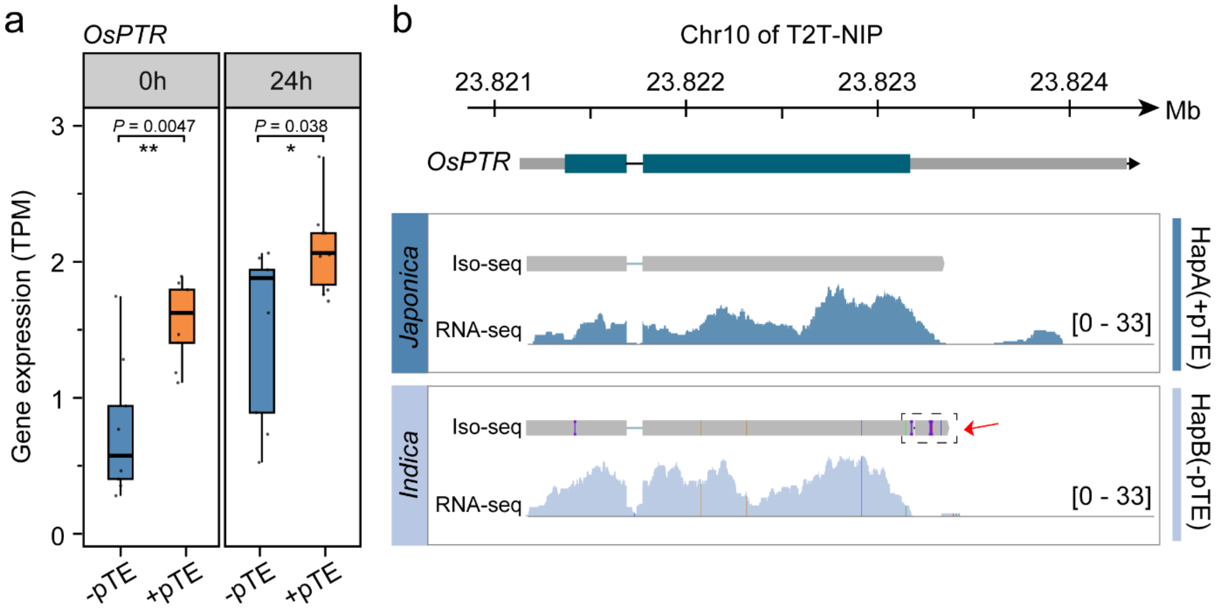

Supplementary Figure 21. Impact of pTE insertion and transcript variants on *OsPTR* expression. a, *OsPTR* expression level in accessions with pTE (+pTE) or without pTE (-pTE). **b**, Iso-seq and RNA-seq reads mapping based on T2T-NIP. The box indicated by the red arrow highlights the difference between the two transcripts. Statistical analysis was performed using a two-tailed Wilcoxon test (*$P < 0.05$, **$P < 0.01$). Source data are provided as a Source Data file.

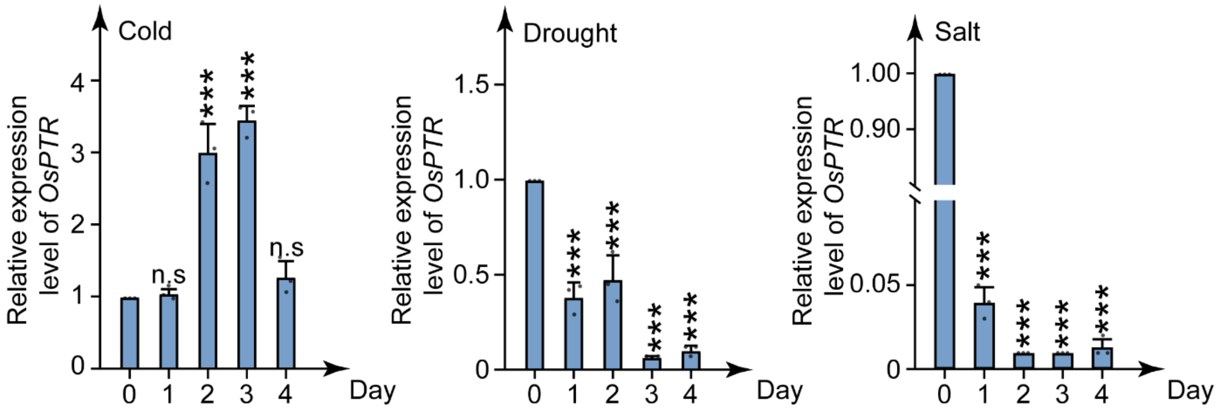

Supplementary Figure 22. Box plot of *OsPTR* relative expression levels over time under cold, drought, and salt stress. Statistical analysis of these data was performed using a two-tailed Wilcoxon test (***$P < 0.001$, ns: $P > 0.05$). Source data are provided as a Source Data file.

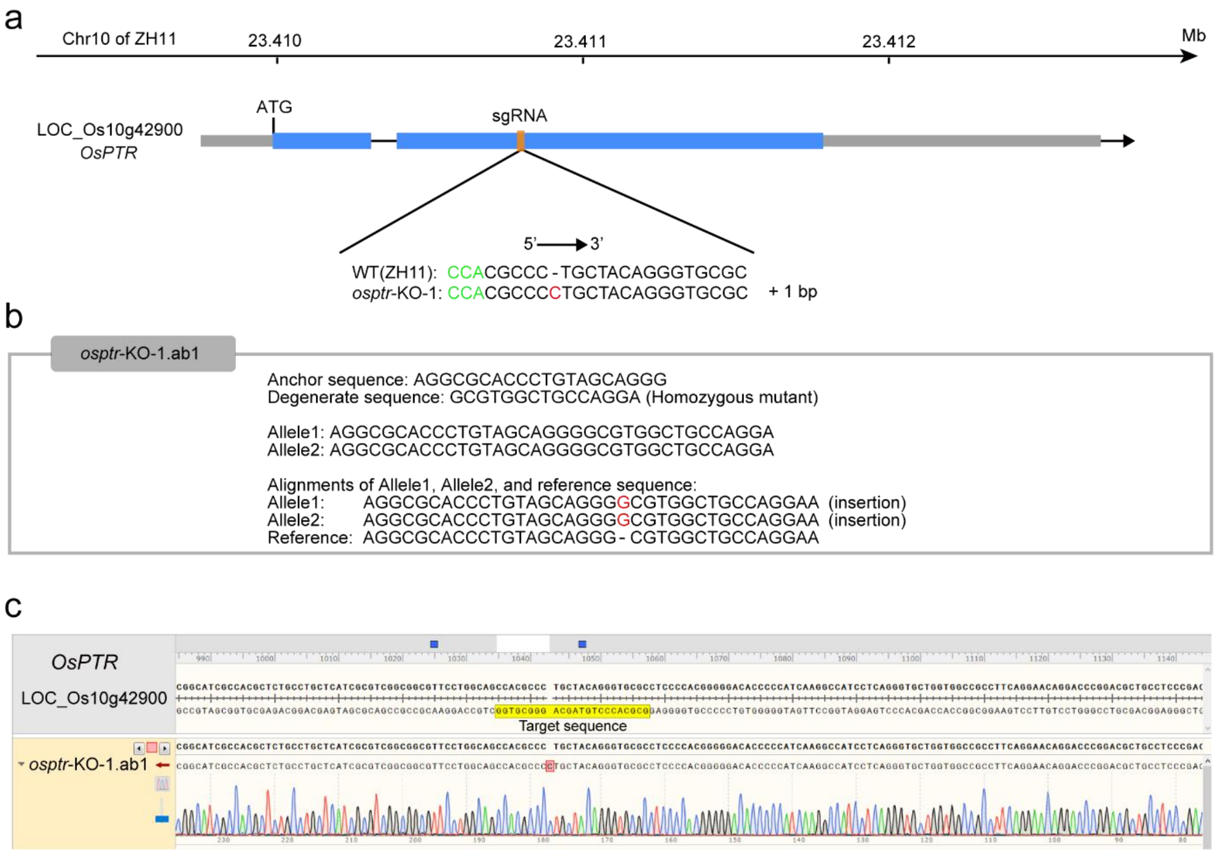

Supplementary Figure 23. Validation of mutation sites in *OsPTR* knockout mutants. a, Target sequences of CRISPR-Cas9 for gene knockout in *OsPTR*. **b**, Homozygous and heterozygous genotyping. **c**, Verifying the knockout target through Sanger sequencing.

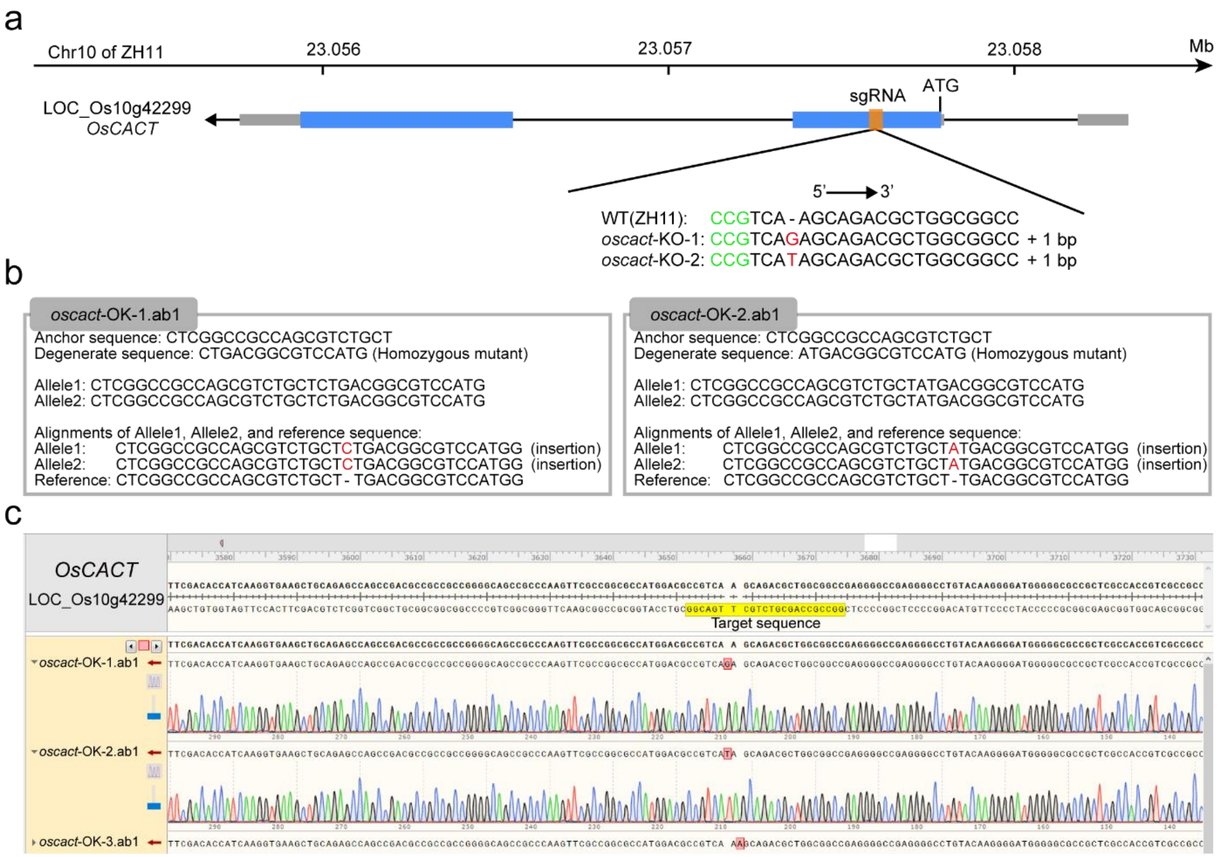

Supplementary Figure 24. Validation of mutation sites in *OsCACT* knockout mutants. a, Target sequences of CRISPR-Cas9 for gene knockout in *OsCACT*. **b**, Homozygous and heterozygous genotyping. **c**, Verifying the knockout target through Sanger sequencing.

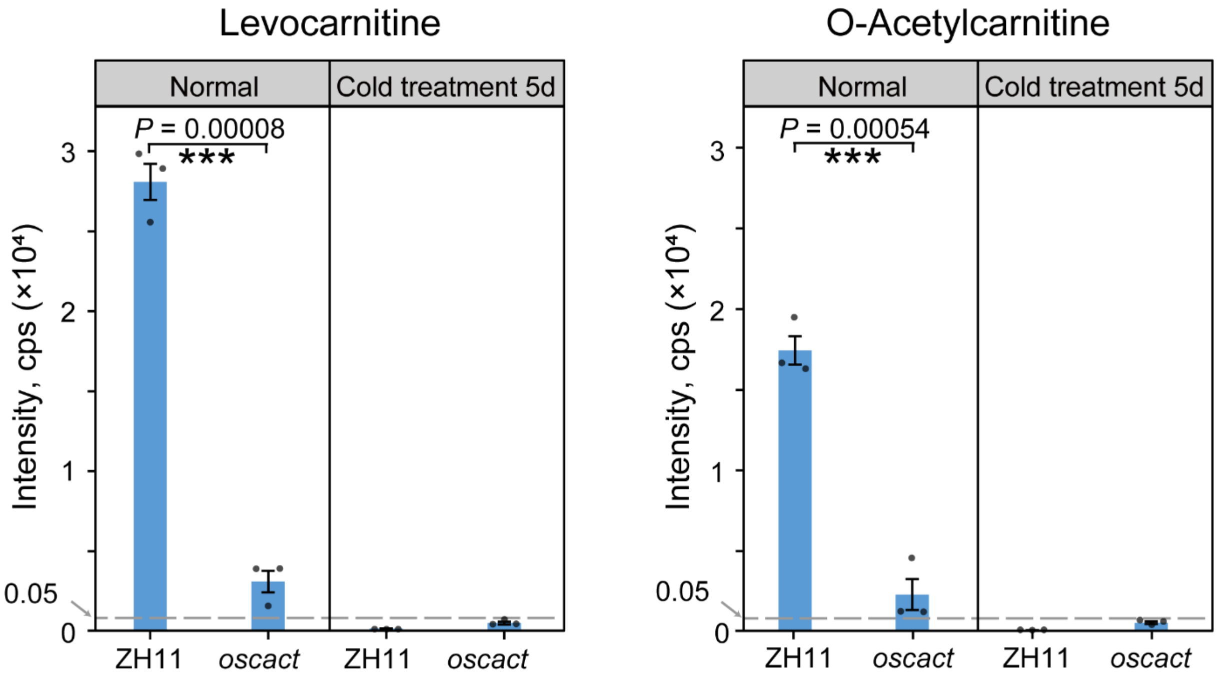

Supplementary Figure 25. The content of Levocarnitine and O-Acetylcarnitine in leaves of ZH11 and *OsCACT* knockout mutants. Statistical analysis was performed using a two-tailed Wilcoxon test (***$P < 0.001$). Source data are provided as a Source Data file.

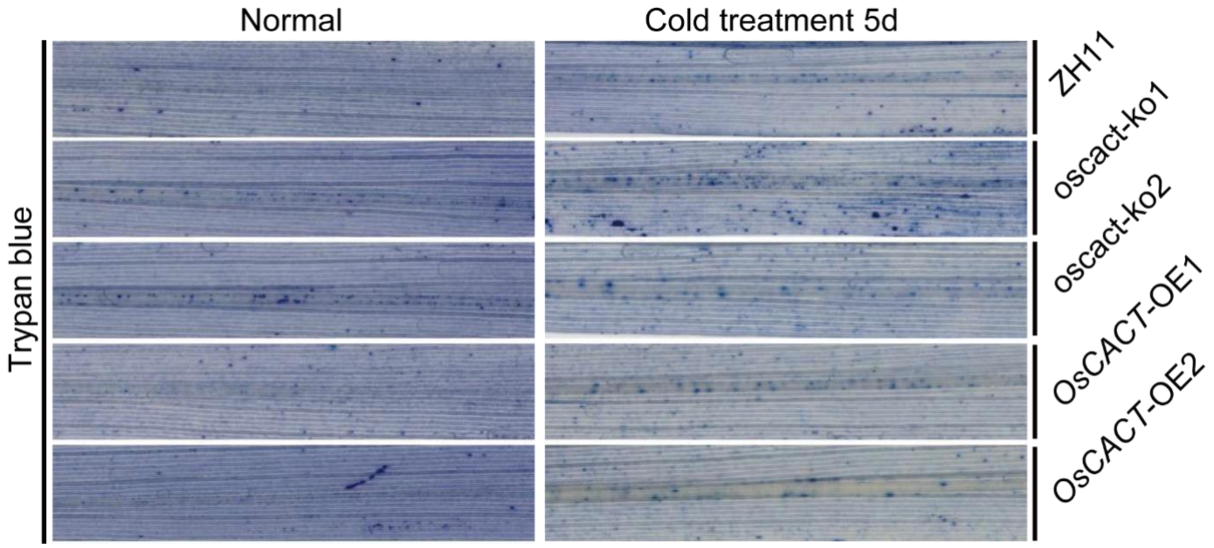

Supplementary Figure 26. Trypan blue staining images of leaves from ZH11, *OsCACT* knockout mutants and *OsCACT* overexpression lines after cold treatment 5 days at four-leaf stage.

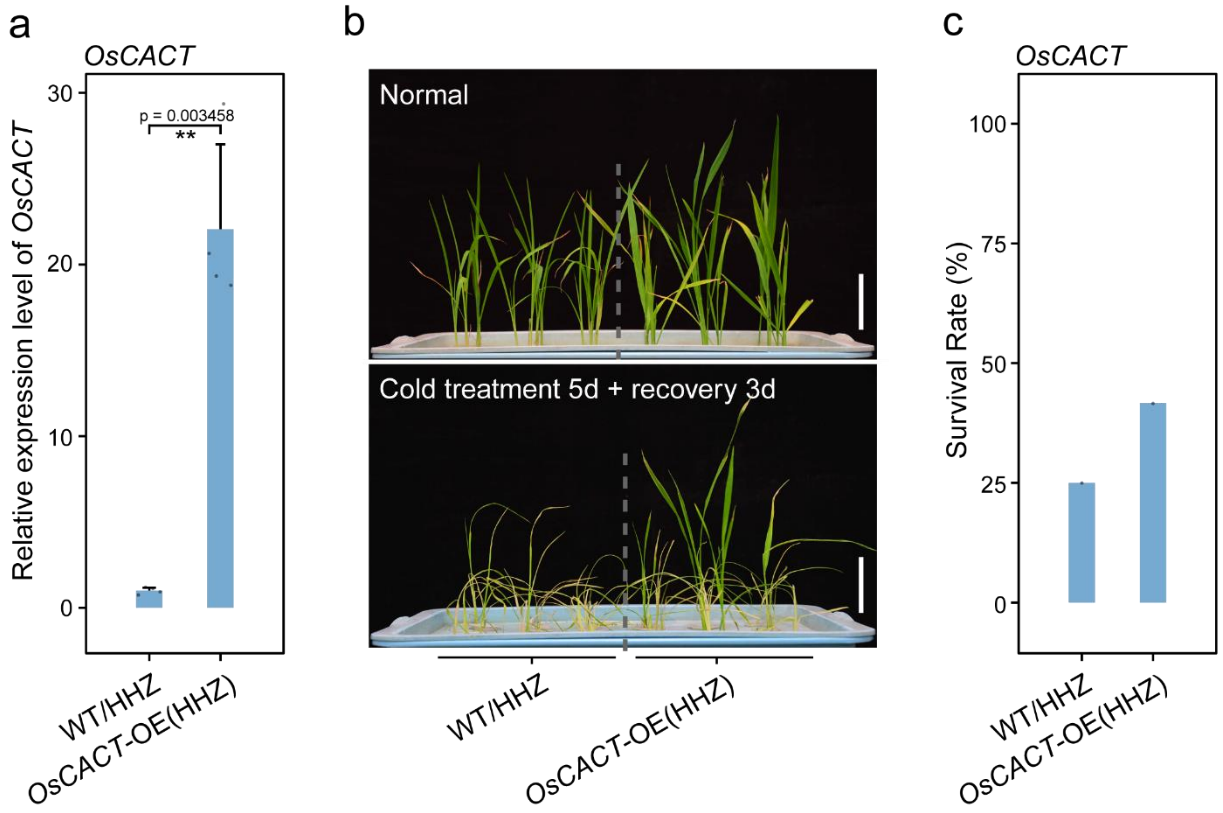

Supplementary Figure 27. Representative images of the *OsCACT* overexpression lines and their corresponding control of HHZ after 5 days of cold treatment followed by 3 days of recovery at the four-leaf stages. a, Expression levels of *OsCACT* in HHZ and overexpression lines. **b**, Phenotypic comparison of HHZ and *OsCACT* overexpression lines before and after cold treatment. **c**, Box plot showing survival rates of HHZ and *OsCACT* overexpression lines before and after cold treatment. Statistical analysis was performed using a two-tailed Wilcoxon test (***P* < 0.01). Source data are provided as a Source Data file.

Supplementary Table 1. Summary of 10 rice accessions.

**Name**	**Accession**	**Subpopulation**	**Country of origin**	**Survival rate (%)**
KOGONI 91-1-1::C1	KO	*indica intermediate*	Mali	0
Koshihikari	K	*temperate japonica*	Japan	100
Lemont	L	*tropical japonica*	USA	52
MADINIKA 1329::GERVEX 8366-C1	M	*indica III*	Madagascar	0
Nanjing11	NJ	*indica I*	China	0
NONA_BOKRA	N	*indica intermediate*	India	0
Nipponbare	R	*temperate japonica*	Japan	100
Fujisaka5	TB	*temperate japonica*	Japan	100
TEQING	T	*indica intermediate*	China	0
9311	Y	*indica II*	China	0

Note: Adapted from table of Zhong *et al*.[1].

Supplementary Table 2. Statistics of sequencing data used for genome assembly.

Accession	Nanopore sequencing data			Illumina sequencing data
	Bases (total)	Reads (total)	Average (bp)	Bases (total)
KO	8,271,054,647	822,765	10,052	15,074,475,600
K	7,064,637,280	560,311	12,608	12,409,729,500
L*	43,860,714,267	2,355,458	18,575	28,178,679,346
M	8,740,446,593	813,871	10,739	12,454,089,900
NJ*	40,741,029,381	2,285,799	18,195	37,228,811,143
N	8,236,413,213	651,034	12,651	11,170,975,500
R	8,379,947,007	753,973	11,114	13,260,237,000
TB	9,201,346,836	663,985	13,857	10,505,896,800
T*	38,184,934,206	2,021,176	18,634	37,997,396,780
Y*	69,306,054,634	4,111,155	16,857	35,422,343,530

Note: The accession number followed by "*" indicates that additional data from public databases[2] has been included.

Supplementary Table 3. Summary statistics of 10 rice genomes.

Accession	Gap number	Gap-free chromosomes	GC percent	TE count	R-AQI	S-AQI	NGS map rate
KO	53	0	0.44	53.73%	97.98	99.75	98.81%
K	50	0	0.44	52.00%	97.90	99.73	99.73%
L	17	3	0.44	52.64%	98.85	98.95	98.82%
M	27	3	0.44	53.60%	98.63	100.00	99.34%
NJ	33	1	0.44	53.90%	98.57	99.49	99.85%
N	41	0	0.44	53.68%	98.31	99.23	99.73%
R	24	1	0.44	52.11%	98.60	100.00	99.73%
TB	16	3	0.44	51.91%	98.94	100.00	99.68%
T	40	0	0.44	53.81%	98.29	99.49	99.81%
Y	37	0	0.44	54.05%	98.33	99.75	99.79%

Supplementary Table 4. Summary statistics of chromosome rearrangement events in 11 rice genomes.

Abbreviation	Insertion	Deletion	Inversion	Translocation	Copy gain	Duplication (gain)	Tandem repeat	Copy loss	Highly diverged regions	Duplication (loss)	Un-aligned region (gain)	Un-aligned region (loss)
T2T-NIP	1,892	2,365	208	6,605	1,185	19,007	377	1,430	4,357	18,243	16,991	17,378
K	1,921	2,363	199	6,585	1,161	17,231	353	1,470	4,588	18,361	16,150	17,529
L	1,846	2,085	185	6,241	1,132	16,109	389	1,302	4,307	17,033	14,931	15,240
R	1,945	2,361	205	6,617	1,176	17,443	366	1,473	4,714	18,301	16,334	17,552
TB	1,914	2,332	208	6,594	1,129	17,076	352	1,404	4,381	18,400	16,313	17,479
KO	838	972	98	3,295	617	10,898	444	717	1,951	9,297	8,656	8,172
M	1,123	1,229	126	4,248	740	12,295	408	825	2,370	11,062	10,034	9,661
N	1,104	1,198	119	4,349	700	12,051	405	813	2,528	11,740	9,963	9,987
NJ	1,009	1,105	122	3,961	674	12,353	387	804	2,285	10,891	9,823	9,305
T	942	1,048	85	3,596	640	11,449	398	750	2,289	10,107	9,123	8,793
Y	795	947	82	3,234	552	11,271	426	664	2,006	9,116	8,912	8,066

Supplementary Table 5. Summary of TEs in 11 rice accessions.

Accession	Length (bp)	Percent	LTR			nonLTR	TIR					MITE		nonTIR	Repeat_region	Intact LTR number	Intact LTR length (bp)
			Copia	*Gypsy*	*unknown*	LINE_element	*hAT*	*CACTA*	*PIF_Harbinger*	*Mutator*	*Tc1_Mariner*	*Tourist*	*Stowaway*	*helitron*			
KO	209,949,234	53.73%	3.04%	19.72%	2.63%	0.06%	1.02%	1.77%	0.51%	4.82%	6.33%	0.27%	0.42%	12.46%	0.52%	21,668	58,565,194
K	194,024,255	52.00%	3.35%	16.96%	2.46%	0.04%	1.10%	1.82%	0.62%	4.66%	6.68%	0.32%	0.43%	12.81%	0.74%	21,773	52,607,729
L	199,715,782	52.64%	3.21%	17.68%	2.89%	0.06%	1.03%	1.79%	0.73%	4.77%	6.47%	0.39%	0.43%	12.68%	0.51%	21,865	54,585,603
M	210,157,719	53.60%	3.06%	19.31%	2.95%	0.04%	1.04%	1.74%	0.52%	5.00%	6.42%	0.28%	0.36%	12.35%	0.53%	21,867	58,841,619
NJ	211,378,094	53.90%	2.72%	20.27%	2.63%	0.07%	1.05%	1.61%	0.58%	4.87%	6.27%	0.23%	0.38%	12.68%	0.54%	21,771	59,935,345
N	207,422,787	53.68%	2.83%	19.64%	2.60%	0.06%	1.03%	1.66%	0.54%	4.90%	6.46%	0.30%	0.44%	12.68%	0.55%	21,526	57,314,177
MH	212,557,498	53.71%	2.98%	20.27%	2.43%	0.04%	1.02%	1.71%	0.51%	4.50%	6.31%	0.30%	0.39%	12.56%	0.67%	21,896	59,387,473
R	195,387,972	52.11%	3.43%	16.38%	2.67%	0.05%	1.09%	1.83%	0.61%	5.21%	6.82%	0.36%	0.44%	12.60%	0.61%	22,030	53,099,744
TB	194,946,034	51.91%	3.36%	16.29%	2.55%	0.04%	1.07%	1.91%	0.61%	4.94%	6.92%	0.37%	0.39%	12.90%	0.58%	21,915	53,048,766
T	210,158,859	53.81%	2.89%	19.76%	2.87%	0.02%	1.07%	1.69%	0.55%	4.96%	6.52%	0.27%	0.42%	12.28%	0.50%	21,547	58,051,523
Y	212,898,567	54.05%	3.09%	19.00%	3.36%	0.02%	0.99%	1.86%	0.57%	5.01%	6.57%	0.28%	0.40%	12.08%	0.81%	21,721	58,851,260

Supplementary Table 6. Summary of GWAS.

Chromosome	Position	ID	Gene symbol	Gene start	Gene end	Type
Chr01	31,145,353	QTL4286	*OsMYL1*	31,056,396	31,057,817	SV
Chr01	40,561,790	QTL5452	*OsCRT3*	40,716,119	40,716,119	TIP
Chr04	32,070,800	QTL19090	*COLD1*	32,171,861	32,176,769	TIP
Chr06	26,910,610	QTL27142	*OVP1*	27,096,620	27,102,686	TIP
Chr07	15,536,138	QTL29610	No genes			TIP
Chr09	1,176,257	QTL35975	*OsDREB6*	13,523,645	13,524,114	SV
Chr12	7,971,788	QTL48039	No genes			TIP

Supplementary Table 7. Sequence and annotation information for the pTE downstream of *OsCACT*.

Chromosome	Start	End	Length (bp)	TE type	Sequence
Chr10	24937,042	24,937,131	90	MITE/*Stowaway*	AGTCAGGGGGTGTTTAGAAACTAGGGACTTATATTTTTGTGAGAGGAACTAAAGTTTAGCCTCACTTTAGTCCCTCCAACCAAACACCAC

Supplementary Table 8. Primers used in RT-qPCR analysis.

Gene ID	Forward sequence (5'-3')	Reverse sequence (5'-3')
Os11g0163100 (*Actin*)	GAGTATGATGAGTCGGGTCCAG	ACACCAACAATCCCAAACAGAG
Os10g0573700	CCTTCAGGAAGATCCTCGCC	CCTAGTGCAGAGCGAGTGAC
Os10g0579800	GAACCTGGTGGTGTACCTGG	GTAGGAGTCGGCGAGGAAAG

Supplementary references

1 Zhong, Y. *et al.* Pan-transcriptomic analysis reveals alternative splicing control of cold tolerance in rice. *Plant Cell* **36**, 2117-2139 (2024).

2 Shang, L. *et al.* A super pan-genomic landscape of rice. *Cell Research* **32**, 878-896 (2022).
