# Supplementary material for: Pangenome analysis of transposable element insertion polymorphisms reveals features underlying cold tolerance in rice
Source: Nat Commun. 2025 Aug 16;16:7634. doi: 10.1038/s41467-025-62887-4 (PMC12357961; doi:10.1038/s41467-025-62887-4)
Supplement: Supplementary file 3 — Description of Additional Supplementary Files [file 41467_2025_62887_MOESM3_ESM.pdf]

Description of Additional Supplementary Files

File Name: Supplementary Data 1
Description: Summary of 165 rice accessions.

File Name: Supplementary Data 2
Description: Summary of accessions for RNA-seq.

File Name: Supplementary Data 3
Description: Enriched GO terms for genes co-expressed with trans-TEs.

File Name: Supplementary Data 4
Description: Information of 138 reported cold-tolerant genes.

File Name: Supplementary Data 5
Description: Summary of interactions between 250 TE-derived lncRNAs and 112 cold-tolerant genes.

File Name: Supplementary Data 6
Description: Information of 54 cold-responsive genes containing pTEs.
