# Supplementary material for: Pangenome analysis of transposable element insertion polymorphisms reveals features underlying cold tolerance in rice
Source: Nat Commun. 2025 Aug 16;16:7634. doi: 10.1038/s41467-025-62887-4 (PMC12357961; doi:10.1038/s41467-025-62887-4)
Supplement: Supplementary file 10 — Reporting Summary [file 41467_2025_62887_MOESM10_ESM.pdf]

nature portfolio

Corresponding author(s): Jia-Ming Song, Ling-Ling Chen, Jijing Luo, Kun Lu

Last updated by author(s): Jul 25, 2025

Reporting Summary

Nature Portfolio wishes to improve the reproducibility of the work that we publish. This form provides structure for consistency and transparency in reporting. For further information on Nature Portfolio policies, see our Editorial Policies and the Editorial Policy Checklist.

Statistics

For all statistical analyses, confirm that the following items are present in the figure legend, table legend, main text, or Methods section.

n/a	Confirmed	
☐	☒	The exact sample size (*n*) for each experimental group/condition, given as a discrete number and unit of measurement
☐	☒	A statement on whether measurements were taken from distinct samples or whether the same sample was measured repeatedly
☐	☒	The statistical test(s) used AND whether they are one- or two-sided<br>*Only common tests should be described solely by name; describe more complex techniques in the Methods section.*
☐	☒	A description of all covariates tested
☐	☒	A description of any assumptions or corrections, such as tests of normality and adjustment for multiple comparisons
☐	☒	A full description of the statistical parameters including central tendency (e.g. means) or other basic estimates (e.g. regression coefficient) AND variation (e.g. standard deviation) or associated estimates of uncertainty (e.g. confidence intervals)
☐	☒	For null hypothesis testing, the test statistic (e.g. *F*, *t*, *r*) with confidence intervals, effect sizes, degrees of freedom and *P* value noted<br>*Give P values as exact values whenever suitable.*
☒	☐	For Bayesian analysis, information on the choice of priors and Markov chain Monte Carlo settings
☒	☐	For hierarchical and complex designs, identification of the appropriate level for tests and full reporting of outcomes
☐	☒	Estimates of effect sizes (e.g. Cohen's *d*, Pearson's *r*), indicating how they were calculated

Our web collection on statistics for biologists contains articles on many of the points above.

Software and code

Policy information about availability of computer code

Data collection	Long-read data were generated by Oxford Nanopore PromethlON. ChIP-seq and BS-seq libraries were sequenced with Illumina Novaseq system.
Data analysis	NextDenovo (v2.5.0), NextPolish (v14.1), RagTag (v2.1.0), BUSCO (v5.4.2), LTR_retriever (v2.9.4), Merqury (v1.3), CentIER (v2.0), Tidk (v0.2.31), MUMmer (v4.0), GenomeSyn (v1.2), SyRI (v1.6.3), EDTA (v2.1), GMAP (v2021.12.17), TBtools-II (v2.001), BPGA (v1.3), minigraph (v0.19), VG (v1.55.0), VCFtools (v0.1.16), PLINK (v2.2.9), ADMIXTURE (v1.3.0), HISAT2 (v2.2.1), SAMtools (v1.21), StringTie (v2.2.0), DESeq2 (v1.18.1), TEtranscripts (v2.2.3), Deeptools (v3.5.6), BWA (v2.2.1), MACS2 (v2.2.9.1), BEDTools (v2.30.0), minimap2 (v2.25-r1173), SQANTI3 (v4.2), ORFfinder, CPC2, PLEK, PLncPRO, RNAplonc, WGCNA, CORNET, TEffectR, Gephi (v0.10.1), GATK (v4.2.6.1), BCFtools (v1.19), EMMAX

For manuscripts utilizing custom algorithms or software that are central to the research but not yet described in published literature, software must be made available to editors and reviewers. We strongly encourage code deposition in a community repository (e.g. GitHub). See the Nature Portfolio guidelines for submitting code & software for further information.

Data

Policy information about availability of data

All manuscripts must include a data availability statement. This statement should provide the following information, where applicable:

- Accession codes, unique identifiers, or web links for publicly available datasets
- A description of any restrictions on data availability
- For clinical datasets or third party data, please ensure that the statement adheres to our policy

The raw sequencing data, genome assembly data and gene annotations generated in this study have been deposited in the National Genomics Data Center (NGDC; https://ngdc.cncb.ac.cn/) under accession number PRJCA032145 [https://ngdc.cncb.ac.cn/bioproject/browse/PRJCA032145]. The previously released transcriptome data and resequencing data used in this study are available at NGDC under accession number PRJCA017960 [https://ngdc.cncb.ac.cn/bioproject/browse/PRJCA017960]. All genome assemblies, annotations and TIP information are available at Rice pTE Database (https://cbi.gxu.edu.cn/RICEPTEDB/). Code used for the pangenome analysis is available at Zenodo (https://doi.org/10.5281/zenodo.15573226).

Research involving human participants, their data, or biological material

Policy information about studies with human participants or human data. See also policy information about sex, gender (identity/presentation), and sexual orientation and race, ethnicity and racism.

Reporting on sex and gender	N/A
Reporting on race, ethnicity, or other socially relevant groupings	N/A
Population characteristics	N/A
Recruitment	N/A
Ethics oversight	N/A

Note that full information on the approval of the study protocol must also be provided in the manuscript.

Field-specific reporting

Please select the one below that is the best fit for your research. If you are not sure, read the appropriate sections before making your selection.

☒ Life sciences ☐ Behavioural & social sciences ☐ Ecological, evolutionary & environmental sciences

For a reference copy of the document with all sections, see nature.com/documents/nr-reporting-summary-flat.pdf

Life sciences study design

All studies must disclose on these points even when the disclosure is negative.

Sample size	We selected 10 rice samples that are geographically diverse and represent a broad spectrum of temperature adaptability for genome assembly and transcriptome analyses. We conducted ChIP-seq and BS-seq sequencing on 2 samples, chosen for their representativeness of indica and japonica rice. For population genomic analyses, a collection of 165 representative rice accessions from a public study was used.
Data exclusions	Data exclusions were based on predefined quality control criteria applied during the annotation of transposable elements and genomic variations, as detailed in the Methods section. Apart from these quality control filters, no other data were excluded from the analyses.
Replication	Three independent biological replicates were performed for the RNA-seq experiments to ensure reproducibility, and all replicates showed consistent results. Additionally, three independent F2 transgenic lines were generated and analyzed to validate the cold tolerance phenotype, with consistent phenotypic outcomes observed across lines.
Randomization	The growth environment, treatment conditions, and growth stages for the RNA-seq samples were kept consistent. Leaves for sequencing were randomly selected from different accessions to avoid bias. For physiological indicator measurements, leaves were randomly collected from ZH11, HHZ, and transgenic lines subjected to the same treatment conditions.
Blinding	Blinding is not applicable for genome sequencing due to their nature. The investigators were blinded to group allocation during collecting data from ZH11,HHZ and transgenic lines.

Reporting for specific materials, systems and methods

We require information from authors about some types of materials, experimental systems and methods used in many studies. Here, indicate whether each material, system or method listed is relevant to your study. If you are not sure if a list item applies to your research, read the appropriate section before selecting a response.

Materials & experimental systems

n/a	Involved in the study
☒	☐ Antibodies
☒	☐ Eukaryotic cell lines
☒	☐ Palaeontology and archaeology
☒	☐ Animals and other organisms
☒	☐ Clinical data
☒	☐ Dual use research of concern
☐	☒ Plants

Methods

n/a	Involved in the study
☐	☒ ChIP-seq
☒	☐ Flow cytometry
☒	☐ MRI-based neuroimaging

Plants

Seed stocks

The plant materials used in this study were described in Supplementary Table 1.

Novel plant genotypes

The OsPTR loss-of-function mutant was constructed using CRISPR-Cas9, with the sgRNA shown in Supplementary Figure 23a. The OsCACT loss-of-function mutant was constructed using CRISPR-Cas9, with the sgRNA shown in Supplementary Figure 24a.

Authentication

Sanger sequencing was used to verify and identify new genotypes, which are described in Supplementary Figure 23c and Supplementary Figure 24c.

ChIP-seq

Data deposition

☒ Confirm that both raw and final processed data have been deposited in a public database such as GEO.

☒ Confirm that you have deposited or provided access to graph files (e.g. BED files) for the called peaks.

Data access links
May remain private before publication.

https://ngdc.cncb.ac.cn/search/specific?db=bioproject&q=PRJCA032145

Files in database submission

T0h-9311_S55_L002_R1_001.fastq.gz
T0h-9311_input_S52_L002_R1_001.fastq.gz
T72h-9311_S56_L002_R1_001.fastq.gz
T72h-9311_input_S53_L002_R1_001.fastq.gz
T72h-Nip_S57 _L002_R1_001.fastq.gz
T72h-Nip_input_S54_L002_R1_001.fastq.gz
TOh-9311_S55_L002_R2_001.fastq.gz
TOh-9311_input_S52_L002_R2_001.fastq.gz
T72h-9311_S56_L002_R2_001.fastq.gz
T72h-9311_input_S53_L002_R2_001.fastq.gz
T72h-Nip_S57 _L002_R2_001.fastq.gz
T72h-Nip_input_S54_L002_R2_001.fastq.gz

Genome browser session
(e.g. UCSC)

https://cbi.gxu.edu.cn/RICEPTEDB/

Methodology

Replicates

One biological replicate was used.

Sequencing depth

All ChIP-seq reads were paired-end 150bp. The number of raw reads and mapped reads were show below:
Sample raw_reads mapped_reads
T0h-9311 47,039,238 44,421,424
T0h-9311_input 46,927,280 46,136,664
T72h-9311 48,001,436 45,788,838
T72h-9311_input 47,497,230 46,610,938
T72h-Nip 47,527,756 45,583,335
T72h-Nip_input 48,138,446 39,086,464

Antibodies

Diagenode C 15410195

Peak calling parameters	MACS2 was used with following parameters "c ${Diagenode}-f BAM -B -g 3.6e8", FDR< 0.05 and P-value< 1e-10.
Data quality	Peak numbers are listed below:<br>Sample Peak numbers<br>T0h-9311 25,807<br>T72h-9311 21,550<br>T72h-Nip 22,816
Software	BWA (v2.2.1), SAMtools (v1.21), MACS2 (v2.2.9.1), BEDTools (v2.30.0)
